# Evaluation of Cochlear Symptoms in Migraine Patients without Vestibular Migraine and/or Ménière’s Disease

**DOI:** 10.3390/audiolres13060084

**Published:** 2023-12-06

**Authors:** Valeria Gambacorta, Giampietro Ricci, Alessandra D’Orazio, Davide Stivalini, Irene Baietta, Vito Enrico Pettorossi, Mario Faralli

**Affiliations:** 1 Department of Medicine and Surgery, Section of Otorhinolaryngology, University of Perugia, 06132 Perugia, Italy; 2 Department of Medicine and Surgery, Section of Human Physiology, University of Perugia, 06132 Perugia, Italy

**Keywords:** cochlear symptoms, fullness, migraine, cochlear migraine

## Abstract

Migraine pathogenic pathways may selectively target the cochlea. A qualitative and quantitative analysis of cochlear symptoms in migraine patients without vestibular migraine and/or Méniere’s disease was conducted. We examined 60 consecutive patients with history of cochlear symptoms, including fullness, tinnitus, and hearing loss. Patients were divided into two groups based on migraine history: M (migraine) and nM (no migraine). The incidence of migraine was compared to a homogeneous control group with dysfunctional and inflammatory dysphonia without cochlear symptoms. The type, time of onset, recurrence, bilaterality of symptoms, and hearing threshold were analyzed. The incidence of migraine was significantly higher (*p* = 0.04) in patients with cochlear symptoms than in the control group. The onset of symptoms is significantly earlier (*p* < 0.05) in the presence of migraine. The fullness, recurrence, and bilaterality of symptoms are associated with migraine in a statistically significant way (*p* < 0.05). Pure tone audiometry shows a statistically significant increase in the hearing threshold (500–1000 Hz) in group M. Based on developing findings, cochlear migraine may be considered as a novel clinical entity, like vestibular migraine. It would be the expression, in the absence of vertiginous symptoms, of a selective suffering of the anterior labyrinth by known operating mechanisms of migraine.

## 1. Introduction

The association between migraine and otoneurological disorders, particularly those related to the vestibular system, has been established for a considerable duration. This connection is supported by epidemiological data, pathogenetic hypotheses, and clinical evidence. Epidemiological studies suggest the association between migraine and recurrent vertigo is not casual, and cannot be attributed solely to the coexistence of these two clinical conditions, which are both highly prevalent [1,2]. Kayan and Hood found a comparable incidence of minor nonspecific balance disturbances in patients with migraine headaches, tension headaches, and a control group without headaches. However, they observed that objective vertigo was significantly more common in migraine patients compared to the rest of the sample (27% vs. 8%) [3]. Numerous efforts have been undertaken to diagnose vestibular migraine [4,5], leading to the establishment of diagnostic criteria by the Barany Society [6,7]. Migraine can cause auditory-related otoneurological disorders. Unlike vestibular disorders, the connection between migraine and cochlear symptoms is complex. While studies have demonstrated that migraine can lead to various audiological symptoms such as fullness, tinnitus, and hearing loss [8,9], further research is required to fully comprehend the impact of migraine on the inner ear. The aforementioned symptoms, which frequently exhibit a fluctuating pattern, are characteristic of the symptom cluster associated with Ménière’s disease [10]. Extensive research in the literature has documented the coexistence of primary headache and hydropic labyrinth disease, as well as the potential occurrence of an overlap syndrome between the latter and vestibular migraine [11].

In summary, given the substantial evidence supporting the relationship between migraine and the vestibular system, which can lead to symptoms such as vertigo and balance disorders, it may be possible for patients with migraine to also experience cochlear symptoms, with or without vertigo, as part of their natural history. The pathogenesis of primary headache involves various mechanisms, such as the release of neuropeptides like Calcitonin Gene-Related Peptide (CGRP) [12], genetic alterations in calcium ion channels [13], and the trigemino-vascular theory [14]. These mechanisms may also lead to auditory disturbances in both central and peripheral auditory pathways, similarly to what occurs in the vestibular system. Therefore, the entirety of the labyrinth could represent an alternative target organ to the algogenic centers mainly involved in primary headache. For this reason, we conducted a quantitative and qualitative analysis of cochlear manifestations in a consecutive population of patients who had no history of vertigo. The study calculated the prevalence of the migraine symptoms in these patients and examined the correlation of migraine with various clinical and anamnestic parameters related to cochlear disorders. These parameters included age of onset, gender, type of symptoms (hearing loss, tinnitus, fullness), fluctuation (recurrence), hearing threshold, and bilaterality of symptoms. The purpose of this study is to verify the possibility of a selective involvement of the auditory system by the pathogenetic mechanisms of migraine and to confirm the existence of cochlear migraine in the absence of vestibular migraine and/or Ménière’s disease.

## 2. Materials and Methods

We examined a consecutive series of patients who presented with reported cochlear symptoms at our center between 7 January and 31 December 2022. The age range of the patients included in the study was 18 to 60 years. The cochlear symptoms considered were fullness, tinnitus, and hearing loss. The meaning of fullness was understood as the feeling of fullness and/or padding in the ears. Tinnitus refers to an auditory perception in the absence of acoustic stimulation, while hypoacusia refers to the actual experience of hearing loss despite the presence of acoustic stimulation. The symptoms mentioned above were assessed when reported either individually or in combination, occurring on one or both sides, and in isolated or recurring episodes. The latter form includes cases with fluctuating symptoms, where the reported symptom is consistently present but varies in intensity.

All patients underwent an audiological assessment, which included pure tone audiometry and impedance audiometry. In cases where further diagnostic definition was necessary, additional investigations were conducted, such as auditory evoked potentials and MRI/Angio of the posterior cranial fossa with and without contrast. Patients with conductive hearing loss, regardless of the cause (inflammatory, dystrophic, or catarrhal), with evidence of audiometric and/or tympanometry alterations were not included in our study. Therefore, those with sensorineural hearing loss or normal hearing were included. Patients with Ménière’s disease, as defined by the diagnostic criteria established by the Barany Society [10], were excluded from the study. Additionally, individuals with a current and/or previous history of other types of vertigo were also excluded. An anamnesis was conducted in each patient to identify any past or current history of migraine headache, following the criteria defined by the International Headache Society (IHS) [15].

Based on the available data, the patients were categorized into two distinct groups. The migraine group (M) consists of patients who experience migraine headaches, with or without aura. The non-migraine (nM) group consists of patients who do not exhibit symptoms of migraine in any of the universally recognized forms. We calculated the prevalence of migraine in the recruited patient population and compared the incidence and relative comparison of cochlear symptoms between female and male migraine patients. We performed the Migraine Disability Assessment Test (MIDAS) [16] on patients in Group M to determine the extent of disability caused by headaches. The obtained data were compared to data from a control group that had dysfunctional and inflammatory dysphonia without cochlear symptoms. The control group was homogeneous in terms of age inclusion. We analyzed the clinical and anamnestic parameters related to the symptoms, including their typology (fullness, hearing loss, tinnitus), time of onset (age), recurrence/fluctuation, bilaterality, and hearing threshold. The incidence of each parameter was calculated and compared between the M and nM groups. The hearing thresholds at frequencies of 500, 1000, and 2000 Hz were considered. The chi square test, with or without Yates’ correction, was used to compare percentage values. The *t*-test was used to compare means and standard deviation. The statistical data were considered significant at *p* values < 0.05, as per standard practice.

## 3. Results

A total of 60 patients (age 44.6 ± 11.2) who met the inclusion criteria were recruited for the study, consisting of 31 males and 29 females. A total of 18 patients (30%) belonged to group M, with 14 females (77.8%) and 4 males (22.2%). The incidence of migraine was 48.2% (14/29) and 12.9% (4/31) in females and males, respectively.

Table 1 presents a summary of the manifestations of migraine and the corresponding level of disability as measured by the MIDAS.

The statistical comparison shows that the percentage incidence of migraine patients with cochlear symptoms is significantly higher in females than in males (the chi square statistic without Yates’ correction was 8.9274: *p* = 0.002; with Yates’ correction, it was 7.3224: *p* = 0.006).

**Figure 1:**
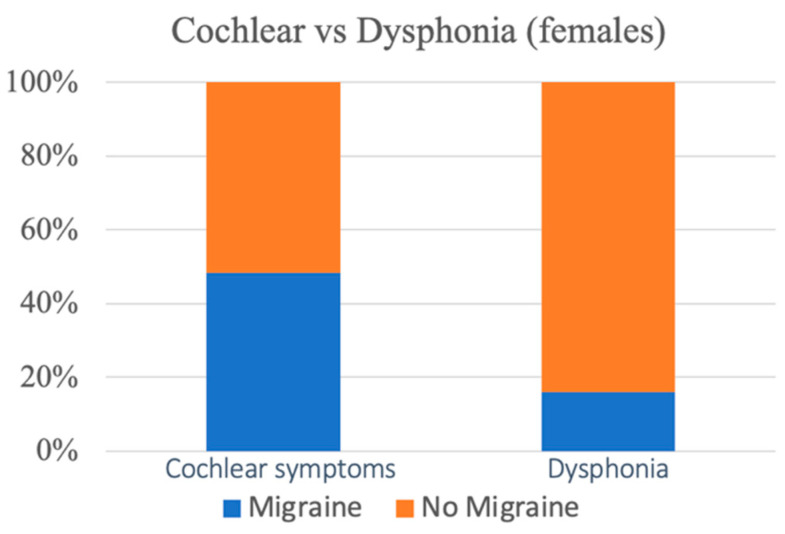
The percentage incidence of female migraine patients with cochlear symptoms is significantly higher compared to female patients in the control group (*p* = 0.0026).

The control group consisted of 45 patients with dysphonia, including 25 females and 20 males with a mean age of 43.3 ± 12.1 years (44.6 ± 11.2 vs. 43.3 ± 12.1: *p* < 0.05). The number of patients belonging to group M was 6 (13.3%), with 4 females (4/25: 16%) and 2 males (10%); the number belonging to the nM group was 39 (86.7%). The incidence of migraine was significantly higher (*p* = 0.04) in patients with cochlear symptoms compared to the control group with hoarseness (chi square statistic: 4.0509). The increase in the incidence of migraine in female patients with cochlear symptoms appears to be more significant compared to female patients with dysphonia belonging to the control group (The chi square statistic without Yates’ correction was 6.2938: *p* = 0.01; with Yates’ correction, it was 4.9252: *p* = 0.026) (Figure 1).

**Figure 2:**
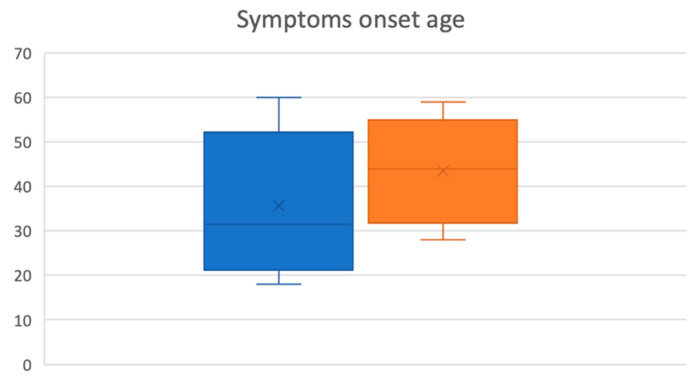
The presence of migraine is associated with an earlier onset of cochlear symptoms (*p* < 0.05). This is illustrated in the graph, where migraine patients (M) are represented in blue, patients without migraine are represented in orange (nM), and the age is displayed in decades on the *y*-axis.

No significant difference (*p* < 0.05) emerges in the ratio between migraine incidence in female vs. male patients belonging to the control group (chi square statistic: 0.3462). The mean age of onset of cochlear symptoms was 34.44 ± 12.25 and 43.79 ± 8.76 in the M and nM groups, respectively. The onset, therefore, is significantly earlier (*p* < 0.05) in the presence of migraine (Figure 2).

Table 2 shows the distribution of cochlear symptoms, individually and variously associated.

**Figure 3:**
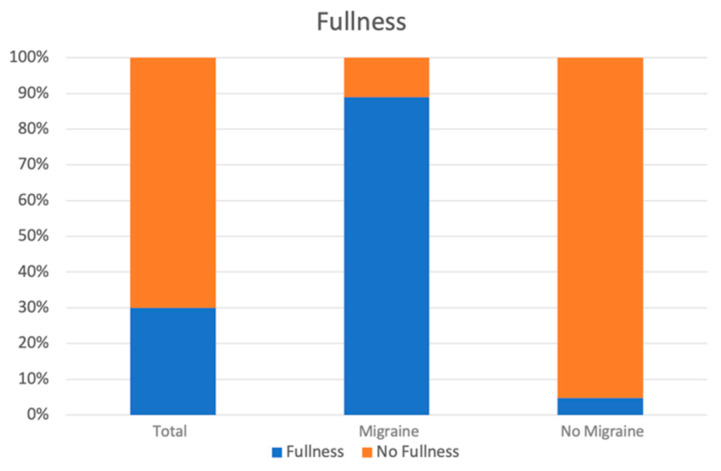
The incidence of the fullness symptom was significantly higher among patients with migraines, as indicated by the graph (*p* < 0.0001).

The most commonly reported symptom was hypoacusis (44 patients), followed by tinnitus (30 patients) and fullness (18 patients). In particular, ear fullness was reported by 16 of 18 patients (88.8%) belonging to group M, and 2 of 42 (4.8%) to group nM. The data are statistically very significant (the chi square statistic without Yates’ correction was 42.4641: *p* < 0.00001. The chi square statistic with Yates’ correction was 38.5525: *p* < 0.0001) (Figure 3).

**Figure 4:**
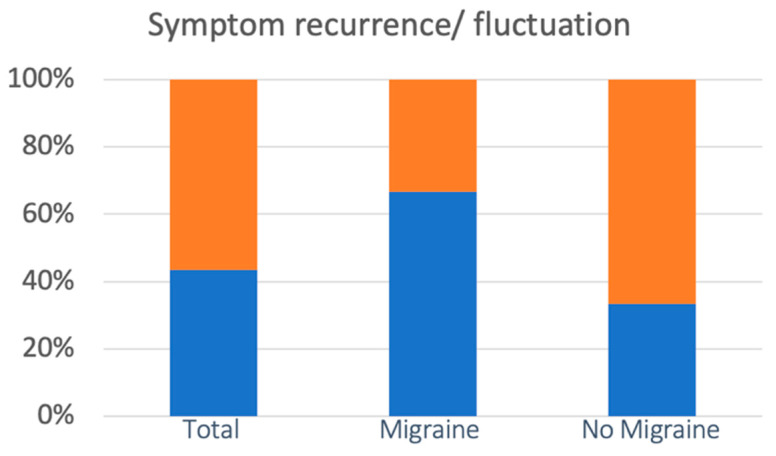
The incidence of recurrent cochlear symptoms was statistically significantly higher in the M group than in the nM group. Cases of recurrence are represented in blue, with cases of no recurrence in orange.

Hearing loss was present in 16 of 18 patients (88.8%) in the M group and in 28 of 42 (67%) patients in the nM group. Although the incidence of hearing loss is higher in group M, this increase is not statistically significant (chi square statistic: 3.1818; *p* = 0.074). However, hypoacusis was found to be temporary in all 16 patients (100%) in the M group and in 8 out of 28 cases (28.8%) in the nM group. The observed difference was statistically very significant (the chi square statistic with Yates’ correction was 17:3: *p* = 0.0001). No significant difference between the groups under examination (M vs. nM) emerged for the tinnitus symptom (*p* > 0.05). A recurrence of symptoms involved a total of 26 patients (43.3%), 12 (66.6%) of 18 patients belonging to the M group and 14 (33.3%) of the 42 patients in the nM group. The incidence of recurrent cochlear symptoms was significantly higher from the statistical point in the M group than in the nM group (the chi square statistic without Yates’ correction was 12.424: *p* = 0.0004; the chi square statistic with Yates’ correction was 10.501: *p* = 0.001) (Figure 4).

**Figure 5:**
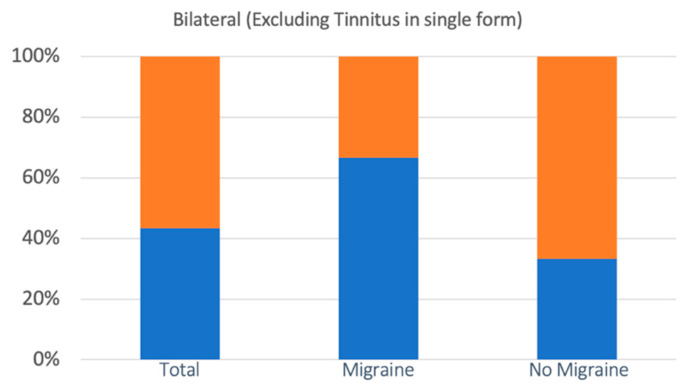
The incidence of bilateral cochlear symptoms (with the exclusion of cases of tinnitus as the only symptom) was statistically significantly higher in the M group than in the nM group. Bilateral symptoms are represented in blue, with no bilateral symptoms in orange.

Bilaterality of symptoms was observed in 28 cases (46.6%), of which 10 concerned the M group and 18 the nM group. The incidence in the two groups was 55.5% (M) and 42.8% (nM), respectively. The increase recorded in group M, however, is not statistically significant (the chi square statistic was 0.8136: *p* = 0.36). In 10 of the 28 cases, the bilaterality involved tinnitus in single form. The remaining 18 cases with one manifestation (hearing loss or fullness), or variously associated (hearing loss, fullness, tinnitus), concerned 12 of the 18 patients of group M (66.6%) and 6 of the 42 patients of group nM (14.2%). In this case, the increase in the incidence of bilaterality in the M group compared to the nM group was statistically significant (The chi square statistic without Yates’ correction was 7.997: *p* = 0.004; the chi square statistic with Yates’ correction was of 6.353: *p* = 0.01) (Figure 5).

Pure tone audiometry highlighted a pathological hearing threshold (>20 dB) with relative sensorineural hearing loss for the three frequencies considered (500–1000–2000 Hz) in 40 patients. Of these, 13 belonged to the M group (13/18: 72.2%) and 27 to the nM group (27/42: 64.2%). No significant difference emerged between the two groups (M vs. nM) in terms of incidence (*p* > 0.05). The mean threshold for the three frequencies was 34.44 ± 10.83 (500 Hz), 32.22 ± 10.74 (1000 Hz), and 29.44 ± 9.06 (2000 Hz) in group M and 25.75 ± 6.56 (500 Hz), 25.95 ± 8.28 (1000 Hz), and 27.14 ± 10.13 (2000 Hz) in the nM group. A statistically significant increase (*p* < 0.05) in the hearing threshold was therefore recorded for the frequencies 500 and 1000 Hz in group M. No statistically significant difference between the two groups concerned the frequency 2000 Hz (*p* > 0.05) (Table 3).

## 4. Discussion

The concept of cochlear migraine was first described in 2018 to investigate the correlation between migraine and auditory impairment. Diagnostic criteria included recurrent or fluctuating unilateral sensorineural hearing loss (low frequency or all frequencies) without vertigo or mild dizziness that does not met the criteria for VM [17]. The initial investigation of the association between migraine and cochlear disorders focused on sudden hearing loss. Viire and Baloh (1996) reported 13 cases of unexplained sudden sensorineural hearing loss (SSNHL) that fulfilled the diagnostic criteria for migraine. They proposed that vasospasm of the cochlear vascular system could be a potential underlying cause [18]. Lee et al. (2000) is the sole source of histopathologic evidence connecting migraine and SSNHL [19]. The case described involved a man with long-lasting migraine who developed SSNHL at the age of 50. The cochlea’s autopsy findings revealed significant fibrosis in the stria vascularis and spiral ligament, indicating a vascular insult. A recent meta-analysis found that migraine is significantly linked to a 1.8-fold higher risk of developing SSNHL [20]. Xue et al. conducted a study to examine and compare auditory outcomes among individuals with migraine, vestibular migraine, and healthy controls.

Research has shown that both the peripheral and central auditory systems are implicated in migraine and vestibular migraine. Additionally, abnormalities in auditory brainstem response (ABR) may serve as an early indicator of dysfunction in the central auditory system [21]. This finding supports a previous study’s conclusion that all migraine patients, including those with normal audiometric results, exhibit ABR abnormalities [22]. Similarly, a study discovered a correlation between migraine and otoacoustic emissions, which examined cochlear function. Migraine patients exhibited decreased otoacoustic emissions (TEAOEs) across different frequencies, despite having normal hearing abilities [23]. The findings obtained from our study indicate that patients who visit our clinic frequently describe cochlear symptoms that are influenced by the migraine. The incidence of migraine appears to be particularly high in the group of patients recruited. The pathogenic mechanisms of migraine and its related clinical manifestations are particularly active in the young adult and middle-aged population, representing the florid phase of the disease [24].

The decision to establish a criterion excluding individuals over the age of 60 is motivated by the need to eliminate the influence of age on hearing threshold and cognitive functions. Additionally, this decision is supported by evidence indicating a gradual decline in the mechanisms involved in the manifestation of migraine among this age group. The statistical significance of the data increases when evaluating the incidence in a population of patients who are homogeneous in terms of age and are affected by dysphonia without cochlear symptoms. In terms of gender, women with cochlear symptoms have a significantly higher prevalence of migraine (M/F, 1:3.5). The data appear predictable given the prevalence of migraine in the general population, which indicates a ratio (M/F) of 1:2–3 [25]. However, it is important to note that the comparison with the control group of patients with dysphonia without cochlear symptoms is particularly significant. In a prior investigation, the male-to-female ratio in vestibular migraine was reported as 1:8 [26]. Consequently, the observations in the cohort of migraine patients with cochlear symptoms would fall within the range between the general population and vestibular migraine. This suggests a greater frequency in females when the labyrinth appears to serve as an additional target organ for the pathogenetic mechanisms of migraine. Additionally, it appears that this action can be selectively performed on the cochlear component of the labyrinth while preserving the vestibular component. This is supported by the lack of vertigo history in the patients we included in our study. In our study, we found that fullness is the cochlear symptom most strongly correlated with the presence of migraine. The data support the outcomes of a prior study, indicating that 54% of patients experiencing fullness met the IHS criteria for migraine. Furthermore, all patients showed symptomatic improvement when using migraine medications, specifically verapamil (82%) or nortriptyline (18%) [27]. As is well known, fullness is a common symptom of hydropic labyrinth disease, often appearing early. Also, there is a recognized comorbidity between Ménière’s disease and migraine, as well as the possibility of an overlap syndrome between Ménière’s disease and vestibular migraine [11].

Recurrent and/or fluctuating fullness in the absence of functional changes in the middle ear may suggest the presence of initial endolabyrinthine hydrops in individuals with migraine. This condition can cause cochlear injury but may not be severe enough to disrupt the homeostasis of endolymph and perilymph, which is responsible for vertigo. Hence, it may be deduced that migraine mechanisms not only tend to support the endolabyrinthic hydropic mechanisms in general, but also contribute to the earlier onset of the latter. Another significant finding of our study pertains to the lower age at which individuals with migraine experience the first signs of cochlear symptoms. This does not seem coincidental if we consider a greater susceptibility of developing MD with earlier onset of symptoms and a greater frequency of bilateral hearing loss when MD patients also had migraine [28]. The central pathogenetic mechanisms that explain the association between headache and vertigo in patients with migraine are widely understood, supporting the existence of a clinical condition known as vestibular migraine. In fact, the vestibular system and the algogenic pathways implicated in migraines share several structures, including the locus coerules, raphe nucleus, and periaqueductal gray [29,30]. During a headache attack, the modulation of these areas can result in different central processing of vestibular information. Neuropeptides, including CGRP (Calcitonin gene-related peptide) [31], and neurotransmitters like 5-hydroxytryptophan and noradrenaline, play a significant role in migraine attacks. These substances, released during an attack, can affect the vestibular nuclei through a receptor system that is widely distributed at this level [32].

Finally, the crucial role of calcium ion channels in regulating the GABA-ergic inhibitory activity of the vestibulo-cerebellum on the vestibular nuclei should not be underestimated. Genetic alterations affecting calcium ion channel subunits have been identified in certain hereditary conditions, including hemiplegic migraine [33,34]. However, these mechanisms do not seem to adequately explain cochlear symptoms to define cochlear migraine as a distinct entity, similarly to what has been observed for vestibular migraine [17]. On the other hand, when considering hypothetical peripheral mechanisms induced by migraine, this definition becomes more convincing in explaining the development of cochlear disorders. Peripheral mechanisms that may explain cochlear symptoms include vascular spasm in the internal auditory artery, leading to transient or persistent ischemia (sudden hearing loss) in the territories supplied by the common cochlear artery, main cochlear artery, or vestibulocochlear artery [18].

Recent laboratory acquisitions on animal models, concerning the trigemino-vascular unit and neurophlogosis mechanisms, have indicated a high probability of the cochlea being involved in migraine processes [35]. In fact, perivascular trigeminal endings have been extensively demonstrated in different regions of the cochlea [36]. In particular, the presence of these structures in the stria vascularis, which is responsible for producing and regulating endolymph, could explain the cochlear symptoms associated with hydropic-like characteristics. The release of vasoactive amines from the trigeminal endings can cause increased vasodilation, permeability, and extravasation, leading to a rise in endolymph production [37]. Auricular fullness, as well as the recurrence (or fluctuation) of symptoms, particularly the fullness itself and hearing loss, whose incidences are significantly higher in migraine patients with cochlear symptoms, are significantly easier to comprehend in light of these recent discoveries. Bilaterality is a further notable characteristic observed in migraine patients. Based on the assumption regarding the pathogenic mechanisms of migraine, it is not surprising that it can cause distress in one cerebral hemisphere leading to unilateral headache, or affect the vestibular system resulting in vertigo, or afflict both hemispheres causing widespread headache and dizziness [38].

Tinnitus does not appear to be influenced by the migraine, contrary to previous findings reported by other researchers [39]. In our study, the symptom was found to be highly prevalent in both groups under consideration, but no statistically significant differences were observed. After a thorough examination of the symptom, it was observed that a substantial portion of our patients who initially presented with tinnitus actually exhibited symptoms of hyperacusis and/or phonophobia. The latter was excluded from the list of cochlear symptoms because it is also a migraine symptom that is included in the diagnostic standards for the definition of headache [15]. This finding may support the counter-trend data observed in our study. The present study did not examine the long-term impact of migraine on the hearing threshold. Some authors have observed a more pronounced decline in the hearing threshold among patients with a prolonged history of migraine [40]. Therefore, we believe it is beneficial to continue studying the patients examined in this article in order to assess the potential long-term progression of audiological symptoms, incorporating techniques such as Transient Evoked Otoacoustic Emissions (TEOAE), Distortion Product Otoacoustic Emissions (DPOAE), and Auditory Brainstem Response (ABR). Our study found a notable rise in the audiometric bone-conducted threshold at frequencies of 500 and 1000 Hz. This increase is likely attributed to the initial hydropic characteristics of hypoacusia, supporting the existing understanding of the association between migraine and Ménière’s disease. Given the limited number of cases and the lack of adequate patient follow-up, additional research is required to better define this final aspect of cochlear function. Recent studies have indicated that patients diagnosed with MD or VM exhibit distinct proinflammatory patterns: Flook et al. conducted a study to examine the cytokine profile of MD and VM in order to differentiate between patients with MD and VM. Their findings suggest that this approach could potentially improve the efficacy of differential diagnosis between VM and MD. This method may also be applicable in the future for accurately distinguishing between patients with Ménière’s disease and patients with cochlear migraine, who do not exhibit vestibular symptoms [41].

### Limitation of the Study

The main limitation of this study is the limited sample size. To validate our findings and test our hypotheses, it would be beneficial to extend our study by increasing the sample size.

## 5. Conclusions

Based on the latest acquisitions and emerging evidence from our study, it is possible to cautiously hypothesize that cochlear migraine may be considered a new clinical entity, similarly to vestibular migraine. It would be the expression, in the absence of vertiginous symptoms, of a selective affliction of the anterior labyrinth caused by known pathogenetic mechanisms associated with migraine. Simultaneously, cochlear symptoms with similar characteristics and influenced by the same underlying mechanisms can manifest as cochlear Ménière’s disease. However, the future progression of this condition into a well-defined disease is still uncertain.

## Figures and Tables

**Table 1:** Migraine manifestations and level of Disability caused by headache in M group.

Patient	Aura	Photophobia/Phonophobia	Days (*)	Intensity (**)	MIDAS Score (***)	MIDAS Grade (****)
1	No	Yes	7	7	10	II
6	No	Yes	6	8	8	II
14	Yes	Yes	9	8	12	III
15	No	Yes	7	7	10	II
16	No	Yes	6	7	9	II
22	No	Yes	8	9	15	III
23	No	Yes	6	7	9	II
27	Yes	Yes	7	8	13	III
28	No	Yes	9	9	18	III
31	Yes	Yes	6	7	9	II
37	No	Yes	9	8	12	III
39	No	Yes	7	7	10	II
41	No	Yes	6	7	9	II
48	No	Yes	9	9	15	III
53	No	Yes	7	8	13	III
54	No	Yes	9	9	18	III
59	No	Yes	6	8	8	II
60	Yes	Yes	9	9	17	III

(*): Headache lasting (days) in the last 3 months. (**): Scale of 0–10, how painful on average the headaches were (where 0 = no pain at all, and 10 = pain as bad as it can be. (***): MIDAS score for each patient. (****): Grade of disability. Grade I: little or no disability (MIDAS score: 0–5); Grade II: mild disability (MIDAS score: 6–10); Grade III: moderate disability (MIDAS score: 11–20); Grade IV: severe disability (MIDAS score > 21).

**Table 2:** The distribution of cochlear symptoms, both individually and in various combinations, as observed in the patients from the two examined groups.

Patient	Hearing Loss	Fullness	Tinnitus	Recurrence	Bilateral		Patient	Hearing Loss	Fullness	Tinnitus	Recurrence	Bilateral
1 (M)	yes	yes	no	no	no		31 (M)	yes	yes	yes	yes	no
2 (nM)	yes	no	no	yes	no		32 (nM)	yes	no	no	no	no
3 (nM)	yes	no	no	no	no		33 (nM)	no	yes	no	no	no
4 (nM)	no	no	yes	no	yes		34 (nM)	yes	no	yes	yes	no
5 (nM)	no	no	yes	no	yes		35 (nM)	yes	no	yes	no	yes
6 (M)	yes	yes	yes	yes	no		36 (nM)	yes	no	no	no	yes
7 (nM)	no	yes	no	no	no		37 (M)	yes	yes	yes	yes	yes
8 (nM)	yes	no	yes	yes	no		38 (nM)	no	no	yes	yes	yes
9 (nM)	yes	no	yes	no	yes		39 (M)	yes	yes	no	yes	yes
10 (nM)	yes	no	no	no	no		40 (nM)	yes	no	no	no	no
11 (nM)	yes	no	yes	no	no		41 (M)	yes	yes	no	no	no
12 (nM)	no	no	yes	no	yes		42 (nM)	yes	no	no	yes	no
13 (nM)	no	no	yes	no	yes		43 (nM)	no	no	yes	no	yes
14 (M)	yes	yes	no	yes	yes		44 (nM)	yes	no	no	no	no
15 (M)	yes	no	no	yes	yes		45 (nM)	no	no	yes	no	yes
16 (M)	yes	yes	yes	yes	no		46 (nM)	yes	no	no	no	no
17 (nM)	yes	no	no	no	no		47 (nM)	yes	no	yes	no	yes
18 (nM)	yes	no	yes	no	yes		48 (M)	yes	yes	yes	yes	no
19 (nM)	no	no	yes	yes	no		49 (nM)	no	yes	no	no	no
20 (nM)	yes	no	no	yes	no		50 (nM)	yes	no	yes	yes	no
21 (nM)	yes	no	yes	no	No		51 (nM)	yes	no	no	no	no
22 (M)	yes	yes	no	no	Yes		52 (nM)	yes	no	yes	yes	no
23 (M)	no	yes	no	yes	Yes		53 (M)	yes	yes	no	no	yes
24 (nM)	yes	no	yes	yes	No		54 (M)	no	yes	no	yes	yes
25 (nM)	yes	no	no	no	No		55 (nM)	yes	no	yes	no	no
26 (nM)	yes	no	no	no	yes		56 (nM)	yes	no	yes	no	no
27 (M)	yes	yes	yes	yes	yes		57 (nM)	no	no	yes	no	yes
28 (M)	yes	yes	no	yes	yes		58 (nM)	no	no	yes	no	yes
29 (nM)	yes	no	no	no	no		59 (M)	yes	no	no	yes	yes
30 (nM)	no	no	yes	yes	yes		60 (M)	yes	yes	no	yes	yes

**Table 3:** Comparison between the average hearing thresholds (dB) in the two groups (M vs. nM) at the frequencies (Hz) examined.

	MdB	nMdB	*p*
500 Hz	34.46 ± 10.83	25.75 ± 6.56	0.0004
1000 Hz	32.22 ± 10.74	25.95 ± 8.28	0.0172
2000 Hz	29.44 ± 9.06	27.14 ± 10.13	0.4

## Data Availability

The data presented in this study are available on request from the corresponding author.
